# 

**Published:** 1890-04

**Authors:** 


CONTENTS:

PAGE

ORIGINAL ARTICLES.—
Contagious Venereal Disease Amongst Horses in Kent County, Canada. By Peter H. Bryce, M.A., M D. . . . 197
The Animal Suture; its Use in Surgery. By Henry O. Marcy, M.D. . . . 215
Report of the Autopsy on the Central Park Chimpanzee. By Dr. Jo M. Linsley, M.D. . . . 230
Lympho-Sarcoma. By S. Brimhall, V.M.D, . . . 234
Notes on the Dentition of the Mexican Hairless Dogs. By James A. Waugh, V.S., U. S. Army, . . . 235
Fibroid Tumor of Shoulder. By Williamson Bryden, V.S. . . . 236
Age of the Horse, Ox, Dog, and other Domestic Animals. By R. S. Huidekoper, M.D., Veterinarian . . . 237

EDITORIAL DEPARTMENT.—
Recognition of American Veterinarians . . . 242
Numbers and Values of Farm Animals in the United States . . . 243

CASES, SELECTIONS, TRANSLATIONS.—
The Action of Iodide of Potash. By Prof. L. Trasbot . . . 246

SOCIETY PROCEEDINGS:
Illinois State Veterinary Medical Association . . . 249
New Jersey State Veterinary Society . . . 250
Pennsylvania State Veterinary Medical Association. . . . 252
Long Island Veterinary Society. . . . 253
The Keystone Veterinary Medical Association. . . . 255
Alumni Association of the American Veterinary College. . . . 256

VETERINARY COLLEGE NOTES:
New York College of Veterinary Surgeons and School of Comparative Medicine . . . 258
American Veterinary College. . . . 258

BOOKS AND PAMPHLETS RECEIVED. . . . 260

